# Improving the Cross-Subject Performance of the ERP-Based Brain–Computer Interface Using Rapid Serial Visual Presentation and Correlation Analysis Rank

**DOI:** 10.3389/fnhum.2020.00296

**Published:** 2020-07-31

**Authors:** Shuang Liu, Wei Wang, Yue Sheng, Ludan Zhang, Minpeng Xu, Dong Ming

**Affiliations:** ^1^Academy of Medical Engineering and Translational Medicine, Tianjin University, Tianjin, China; ^2^College of Precision Instruments and Optoelectronics Engineering, Tianjin University, Tianjin, China

**Keywords:** brain–computer interface, electroencephalography, rapid serial visual presentation, event-related potential, cross-subject

## Abstract

The brain–computer interface (BCI) is a system that is designed to provide communication channels to anyone through a computer. Initially, it was suggested to help the disabled, but actually had been proposed a wider range of applications. However, the cross-subject recognition in BCI systems is difficult to break apart from the individual specific characteristics, unsteady characteristics, and environmental specific characteristics, which also makes it difficult to develop highly reliable and highly stable BCI systems. Rapid serial visual presentation (RSVP) is one of the most recent spellers with a clean, unified background and a single stimulus, which may evoke event-related potential (ERP) patterns with less individual difference. In order to build a BCI system that allows new users to use it directly without calibration or with less calibration time, RSVP was employed as evoked paradigm, then correlation analysis rank (CAR) algorithm was proposed to improve the cross-individual classification and simultaneously use as less training data as possible. Fifty-eight subjects took part in the experiments. The flash stimulation time is 200 ms, and the off time is 100 ms. The P300 component was locked to the target representation by time. The results showed that RSVP could evoke more similar ERP patterns among subjects compared with matrix paradigm. Then, the included angle cosine was calculated and counted for averaged ERP waveform between each two subjects. The average matching number of all subjects was 6 for the matrix paradigm, while for the RSVP paradigm, the average matching number range was 20 when the threshold value was set to 0.5, more than three times as much larger, quantificationally indicating that ERP waveforms evoked by the RSVP paradigm produced smaller individual differences, and it is more favorable for cross-subject classification. Information transfer rates (ITR) were also calculated for RSVP and matrix paradigms, and the RSVP paradigm got the average ITR of 43.18 bits/min, which was 13% higher than the matrix paradigm. Then, the receiver operating characteristic (ROC) curve value was computed and compared using the proposed CAR algorithm and traditional random selection. The results showed that the proposed CAR got significantly better performance than the traditional random selection and got the best AUC value of 0.8, while the traditional random selection only achieved 0.65. These encouraging results suggest that with proper evoked paradigm and classification methods, it is feasible to get stable performance across subjects for ERP-based BCI. Thus, our findings provide a new approach to improve BCI performances.

## Introduction

Brain–computer interfaces (BCIs) are communication systems that allow people to send information to a computer or commands to other electronic devices by only measuring brain activities, without requiring any peripheral (muscular) activity (Wolpaw et al., 2002). Compared with all kinds of BCIs, the event-related potential (ERP)-based BCI has made great progress and achieved exciting results. However, the process from laboratory to application has encountered bottlenecks, facing three cross-problems, which are cross-subject, cross-time, and cross-scene. Because of individual differences, it takes a lot of time and efforts to calibrate the traditional BCI system when it is applied to new subjects. As an effective paradigm, the Rapid Serial Visual Presentation (RSVP) can extract stable BCI specific features and strive to achieve high reliability cross-subject BCI technology, which can be used to expand the BCI approach to enable high throughput target image recognition applications (Sajda et al., 2003; Gerson et al., 2006; Bigdely-Shamlo et al., 2008). Using electroencephalography (EEG) signals to label or rank images is of practical interest, because many kinds of images cannot be automatically labeled or ranked by computers (Gerson et al., 2006). A common way here is to use an EEG speller system, which is a typical BCI system, by performing a clever paradigm to induce specific ERP components (e.g., P300 component).

Nowadays, most EEG speller systems were based on a modified P300 speller. The idea was that the participant was unaware when a target stimulus would appear; hence, its presentation on screen elicited the P300 reflecting the orientation of the participant’s attention to the stimulus. Furthermore, the P300 component exhibited significant waveform characteristics in the time domain. It was essential to optimize the P300 detection algorithm because it determined the reliability and accuracy of BCI systems. Thus, there has been promising analytical frameworks proposed to leverage advanced signal processing, independent component analysis (ICA) (Bigdely-Shamlo et al., 2008), xDawn (Rivet et al., 2011), common spatial pattern (CSP) (Ramoser et al., 2000), hierarchical discriminant component analysis (HDCA) (Sajda et al., 2010), sliding HDCA (sHDCA) (Marathe et al., 2013), and convolutional neural network (CNN) (Cecotti et al., 2014) with the attempt to detect P300 signals as well as to later develop high performance on ERP-based BCI. The first BCI speller based on P300 was the alphabet speller system (FD speller) proposed by Farwell and Donchin (1988). The characters (26 letters and other control characters) were arranged in a 6 × 6 matrix, and the user had to focus attention to the target symbol while the rows and the columns were intensified one by one in random order. When the locked symbol was hit by rows or columns (probability is 1/6), it induced a P300 component. Guan et al. convinced that if the possibility of a character being hit is small, then the P300 component induced was evident and the detection is easier (Guan et al., 2004), then they created and developed a single-character display random flashing speller system (SC speller), which was based on the FD speller (the probability that the character was hit was 1/36). By averaging several repeats of stimuli, the enhanced P300 could be detected and the letters written by the user can be inferred. Many changes of the Matrix Speller had been studied to improve communication rate and stimulation performance. Furthermore, Townsend et al. (2010) proposed a paradigm speller system-based checkerboard, in which random flash hit multiple characters, and then a special coding was used to determine the expected characters of the subject. Roark et al. (2013) proposed Huffman scanning, which used Huffman coding to select the highlighted symbols, which were based on the FD speller, which achieved higher accuracy and mean bit rate.

However, as we all know, the process of BCI from laboratory to application has encountered bottlenecks, facing three cross-problems, which are cross-subject, cross-time, and cross-scene. Because of individual differences, it takes a lot of time and efforts to calibrate the traditional BCI system when it is applied to new subjects. Moreover, a major challenge in BCIs is that different subjects have different neural responses to the same stimulus, and even the same user can have different neural responses to the same stimulus at different time and locations (Cecotti and Graser, 2011; Yin et al., 2013; Chen et al., 2014). Besides, when calibrating the BCI system, acquiring a large number of subject specific labeled training examples for each new subject is time consuming and expensive (Marathe et al., 2016; Chen et al., 2019a, b).

In the past two decades, amounts of the research on cross-subject BCI achieved exciting achievements, while also many problems to be solved. Regarded as an important part of cross-subject BCI study, scholars had made big efforts in searching suitable paradigms that could reduce individual differences in EEG signals. Their works confirmed that both FD speller-based recognition accuracy (Brunner et al., 2010) and matrix-based speller had advantage in intersubject study, but the results also showed that different subjects had obvious differences in EEG signals. Besides, Acqualagna and Blankertz (2013) compared rapid serial visual presentation (RSVP) to other matrix-based spellers, excitingly found that the results showed a mean online spelling rate of 1.43 symb/min and a mean symbol selection accuracy of 94.8% in best condition, indicating the promising application of RSVP for intersubject and also cross-subject BCI study (Bigdely-Shamlo et al., 2018). Coincidentally, Bigdely-Shamlo et al. got an average accuracy of 79% using the RSVP paradigm in the cross-subject BCI study of 10 subjects’ classification, but there were still some place for improvement (Acqualagna and Blankertz, 2013). Apart from paradigms, feature extraction also plays an important role in BCI performance, and some work had sought many ways to optimize features. In order to improve the BCI performance, Zhang et al. (2009) proposed feature selection strategies based on principal component analysis and genetic algorithms and achieved an average precision of 0.72. Besides, Kadioglu et al. (2018) designed an iterative algorithm to yield robust mean vector and covariance matrix estimates, and then applied eigen analysis on the estimated robust covariance matrix. The result was a principled way of dealing with outliers in simple data that was, yet achieved the AUC value of 0.75 in cross-subject BCI (Kadioglu et al., 2018). As for the pattern recognition, some algorithms (Gordon et al., 2017; Hajinoroozi et al., 2017; García-Salinas et al., 2019; Fernandez-Rodriguez et al., 2020), such as genetic algorithm and transfer learning (Huang et al., 2011, 2017; Jalilpour et al., 2020), were applied to improve the accuracy and speed of the spellers. On the other hand, the best AUC performance was still not higher than 0.75 (Krusienski et al., 2006; Koçanaoğulları et al., 2020), and most works were still based on small samples. Moreover, Zeng et al. (2019) proposed an authentication method based on hierarchical discriminant component analysis (HDCA) and genetic algorithm (GA) and got the averaged accuracy of 88.88% in also a small sample of 15 subjects. Above all, an appropriate way about cross-subject BCI performance in a large sample still needs to be solved. Therefore, improving the performance of the cross-subject BCI under the condition of establishing model data was an extremely meaningful subject of the study.

In this study, 58 subjects took part in the experiments, and RSVP was employed as the evoked paradigm, and then correlation analysis rank (CAR) algorithm was proposed to improve the cross-subject classification and simultaneously use as less training data as possible. Then three aspects of BCI performance, including ERP waveform, ITR, and ROC, were computed to compare with the traditional methods.

## Materials and Methods

### Participants

Fifty-eight participants (29 males, 29 females, age 20–30, mean = 25.17, *SD* = ± 8.4) took part in the experiment. All had normal or correct-to-normal visual acuity and all without any history of neurological disease or injury. None of them reported a history of psychiatric, neurological, or other serious diseases, which might have affected the experimental results. Before the experiment, the experimental procedure was fully explained to each participant, and all participants signed written consent forms. The experiment was conducted with approval from the Academy of Medical Engineering and Translational Medicine, Tianjin University.

### Apparatus

**FIGURE 1:**
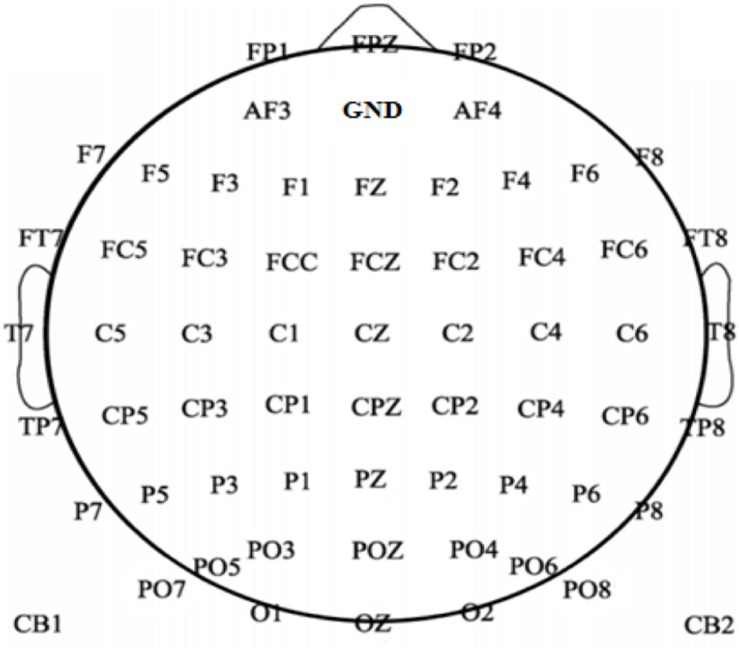
Electrode positions; 64 channels (FPZ, FP1,2, AF3,4, FZ, F1-8, FCZ, FC1-6, FT7,8, CZ, C1-6, T7,8, CPZ, CP1-6, TP7,8, PZ, P1-8, POZ, PO1-8, OZ, O1,2, CB1,2) were collected during the experiment. All the electrodes were referenced to the right mastoid using a forehead ground.

EEG was recorded at 2000 Hz using Neuroscan’s Scan 4.5 system with 64 electrodes (FPZ, FP1,2, AF3,4, FZ, F1-8, FCZ, FC1-6, FT7,8, CZ, C1-6, T7,8, CPZ, CP1-6, TP7,8, PZ, P1-8, POZ, PO1-8, OZ, O1,2, CB1,2), and the EEG equipment was set a 50 Hz notch filter to eliminate the power line interference during acquisition. The electrode position was placed in accordance with the international standard 10–20 electrode system. All the electrodes were referenced to the right mastoid, using a forehead ground, as shown in Figure 1. The impedance of every electrode was kept below 5 kΩ. The RSVP character flashing stimulus interface was written by using the Psychtoolbox software. Stimuli were presented on a 24″ TFT screen with a refresh rate of 120 Hz and a resolution of 1920 × 1280 p × 2. Data analysis and classification were performed with MATLAB and Python. Furthermore, data preprocessing used EEGLAB in the MATLAB environment.

### Experimental Paradigm

The principle of the RSVP paradigm applied to the BCI spelling device was that all symbols were presented one by one in a random order on the screen center in front of the subjects. Previous studies had shown that different characters that presented with different colors had better inducing effects (Farwell and Donchin, 1988). Different colors allowed subjects to better distinguish among characters. On the basis of using colorful letters, the randomness was changed to sequential presentation in order to increase the recognition ability of the subjects in this study.

**FIGURE 2:**
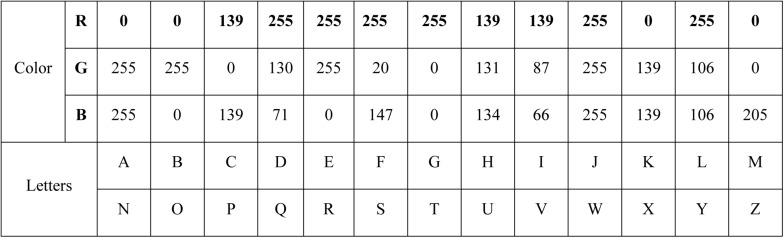
The color of each letter.

Letters’ color and RGB values were shown in Figure 2. As can be seen, the principle of character color selection in this experiment was to improve the arousal of characters to subjects as much as possible and to consider the differences of color senses among different subjects. In order to keep the experiment fair and just possible, the main principles were as follows. First, the span of letters’ color needs to be bigger. Second, the colors of adjacent characters were not the same color system, for example, light blue and dark blue, so as not to affect the subjects’ cognition. Third, too much color for visual stimulation in the black background was not chosen, which avoided the appearance of the visual shadow to the subjects in the experiment and affected the effect of the experiment. Fourth, the character color should flicker randomly in order to show fairness to all subjects.

**FIGURE 3:**
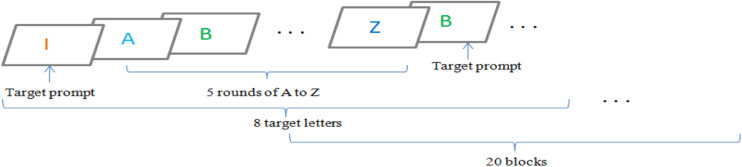
The image-based RSVP framework for experiment.

**FIGURE 4:**
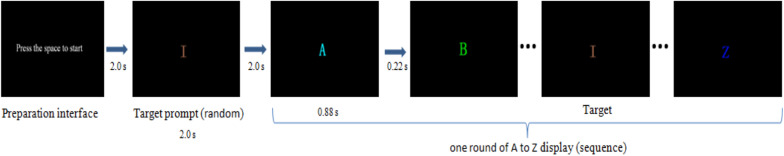
The RSVP paradigm in one round.

The diagram of the experiment is shown in Figures 3, 4; as can be seen, different letters were used as stimulus, and the interval time was 100 ms. Participants sat in a comfortable environment and kept the distance they felt comfortable without light and other interference. First, the preparation interface appeared on the screen. When the participant was calm and remained in a calm state, “Press Space to Start” was clicked. Then there was an RSVP interface. The displayed characters were 26 English capital letters. The target letter was out of order. After it appeared, the letters were arranged in the order from A to Z. After A to Z was proposed, the next round would appear. For a target letter, there are a total of five rounds. Each block contained eight target letters. Each experiment consisted of 20 blocks. In each round, the subjects were asked to count the number of times in which the target occurred fast and quietly. Each subject was required to carry out three experiments with a time interval of 2 days. Before the first experiment, the subjects must go through certain training so that they could be familiar with the contents of the experiment and achieve better results in the next experiment. The number of training tests varied from person to person with the average of 16 rounds.

In summary, each participant did three experiments, with a time interval of 2 days. Each section consisted of 20 blocks, and each block consisted of 8 trials, and each trial consisted of 5 rounds.

## Data Analysis

### EEG Data Processing

The EEG data were re-referenced by averaging both ears and were then filtered by a low-pass filter with a 0.1–30 Hz bandpass. The data were downsampled from 2000 to 200 Hz, and each symbol was divided into epochs ranging from −200 to −800 ms for further analysis.

Therefore, the resulting matrix was 64 × 200 (64 EEG channels and 200 time samples). In the experiment, the EEG equipment was set a 50 Hz notch filter to remove the interference from power frequency noise during acquisition, and we used a 0.1–30 Hz bandpass filter to remove high-frequency noise in EEG data preprocessing, which disturbed the result greatly. In this experiment, we selected the channel data (FZ, CZ, PZ, O1, O2, OZ; Marathe et al., 2016) related to visual stimuli in order to analyze.

### Coherence Average ERP Signals

The data that belong to each person’s EEG induced by the experimental paradigm range from −200 to −800 ms based on the set of the stimulation. We select the data of 200 ms (−200∼0 ms) as the baseline correction and then independently average all previous times of target and nontarget, respectively, so as to obviously see their grand-average ERPs.

### Processing Method

#### Coherent Average Algorithm

Coherent average algorithm is one of the most popular methods to extract weak signals in strong noise background. American scholar Dawson overlapped different EEG patterns on the same paper to obtain the initial EEG waveforms. Because the P300 signal studied in this paper is strictly time-locked, the background noise can be assumed as a random signal that is different from stimulus, so this method can be used in drawing the time domain waveform.

We think *x*_i_(*m*) represents the pretreated EEG signals, *s*(*m*) represents the P300 signals, and *n*_i_(*m*) represents the background noise, so:

(1) $$x_{j}\left(m\right)=s\left(m\right)+n_{j}\left(m\right),j=1,2,\ldots M$$

where *j* represents the trials, *M* represents the number of trials, and *m* represents the value of each sampling point. The estimated P300 signals can be obtained by averaging the preprocessed signals *M*:

(2) $$\overset{⌢}{s}\left(n\right)=\frac{1}{M}\sum_{i=1}^{M}x_{\text{i}}\left(n\right)=s\left(n\right)+\frac{1}{M}\sum_{i=1}^{M}n_{\text{i}}\left(n\right)$$

By the random characteristics of noise, the above can be ordered as $\frac{1}{M}\sum_{i=1}^{\text{M}}n_{\text{i}}\left(n\right)=0$. Therefore, the superposition process can enhance the P300 signal effectively and draw an obvious and smooth P300 waveform.

#### Angle Cosine Similarity

Angle cosine similarity is evaluated by calculating the angle cosine value of two vectors. The angle cosine similarity draws the vector into the vector space according to the coordinate value.

For a two-dimensional space, it is clear from the vector dot product formula that:

(3) $$\cos\theta =\frac{a•b}{∥a∥∥b∥}$$

Assuming the coordinates of the vector a, b are (*x*_1_,*y*_1_) and (*x*_2_,*y*_2_):

(4) $$\cos\theta =\frac{x_{1}x_{2}+y_{1}y_{2}}{\sqrt{x_{1}^{2}+y_{1}^{2}}\times \sqrt{x_{2}^{2}+y_{2}^{2}}}$$

The value of cos⁡θ ranges from -1 to 1, and the larger the value of cos⁡θ, the smaller the angle between the vectors, which means they have more similarity.

#### Information Transfer Rate

In addition to the usual criterion, namely the classification accuracy, the performance of the proposed system was evaluated by the information transfer rate (ITR). The ITR is an important criterion to determine the system performance in BCI studies, especially P300 speller protocols.

It shows the amount of information that can be transferred in a minute (Krusienski et al., 2006) and is defined as:

(5) $$ITR=\frac{\log_{2}N+P\log_{2}P+\left(1-P\right)\log_{2}\frac{1-P}{N-1}}{T}$$

where N denotes the number of classes, P is the accuracy of classification, and T indicates the time interval that character selection is performed in minute.

#### Linear Discriminant Analysis

As we all know, there are many classification methods for EEG signals, which are mainly divided into two types: linear classification method and nonlinear classification method. The selection problems of these two types have been controversial (Muller et al., 2003). Nonlinear classifiers such as support vector machines (SVMs) (Gehler and Schölkopf, 2002) can obtain higher classification accuracy than linear classifiers on some data, but the algorithm modeling process is complicated, in which it is difficult to select suitable kernel functions, and it is sensitive to the change of subjects and is not suitable for cross-subject classification studies. The linear classifier model can be learned quickly and has strong generalization ability, and the classification effect is good enough, which may be more suitable for cross-subject BCI research. Linear discriminant analysis (LDA) (Xanthopoulos et al., 2012) has a good application effect in the field on BCI based on P300.

Offline classification used the LDA for distinguishing target and nontarget letters. LDA is a dimensional reduction technique for monitoring learning, which means that each sample of its data set is printed. Projecting the data onto a low dimension, one expects the projection points of each category of data to be as close as possible, and the greater the distance between the classification centers of the different categories of data, the better the results. The LDA algorithm has high classification accuracy. According to the research content of this paper, the data of subjects and the cross-subject data are classified and calculated perfectly.

#### Correlation Analysis Rank (CAR) Algorithm

The similarity of matching with the new individual refers to the similarity calculation of the ERP waveform, so there is strongest similarity with the tested individuals in the first place. The most commonly used method to measure the correlation between two variables is to calculate the Pearson correlation coefficient, which can characterize the correlation between two linear variables. The formula is as follows:

(6) $$r=\frac{1}{n-1}\sum_{i=1}^{n}\left(\frac{X_{\text{i}}-\bar{X}}{s_{X}}\right)\left(\frac{Y_{\text{i}}-\bar{y}}{s_{y}}\right)$$

We think *r* represents the correlation coefficient, which describes the linear correlation degree between the two variables; n stands for the sample size; *X*_i_ *Y*_i_ represents the two variables, and represents the observed values of the two variables, respectively; and $\bar{X}$ $\bar{Y}$ represents the mean value of the two variables, respectively.

In this study, the correlation between each subject and the other subjects was calculated and the correlation coefficient was sorted, which was used as the standard for the number of subjects in the training set.

## Results

### ERP Analyses

**FIGURE 5:**
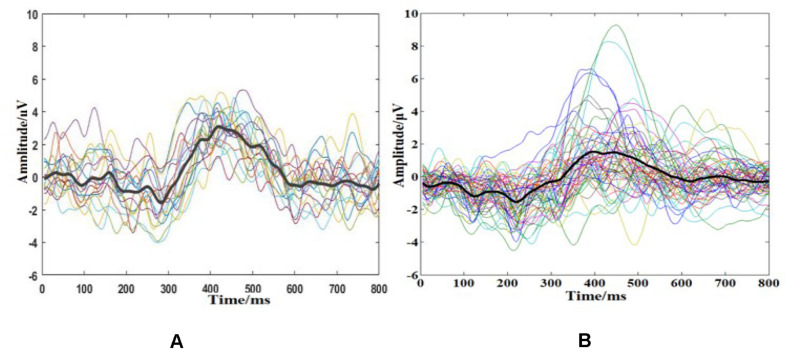
Target ERP waveforms **(A)** of the same subject from 20 sessions and **(B)** those from 58 different subjects. In **(A)**, all lines were from one subject, and each line presented ERP waveform extracted from one session; the black lines represented the average ERP waveforms across all sessions. In **(B)**, each line presented ERP waveform extracted from one subject averaged over 20 sessions, and the thick lines were the mean values across all subjects.

ERP waveforms were drawn using a coherent average algorithm, the target ERPs of the same subject from different sessions are shown in Figure 5A, and the averaged ERPs from different subjects are depicted in Figure 5B. As can be seen, target ERP waveforms from one subject were more similar, in either amplitude or latency, but individual difference could be obviously observed in Figure 5B. The large EEG differences between individuals make it more difficult to realize the high performance on cross-subject BCI system, and the similar BCI-specific characteristics between individuals make it possible to have a wide application in universal BCI.

### Cosine Similarity Analysis

**FIGURE 6:**
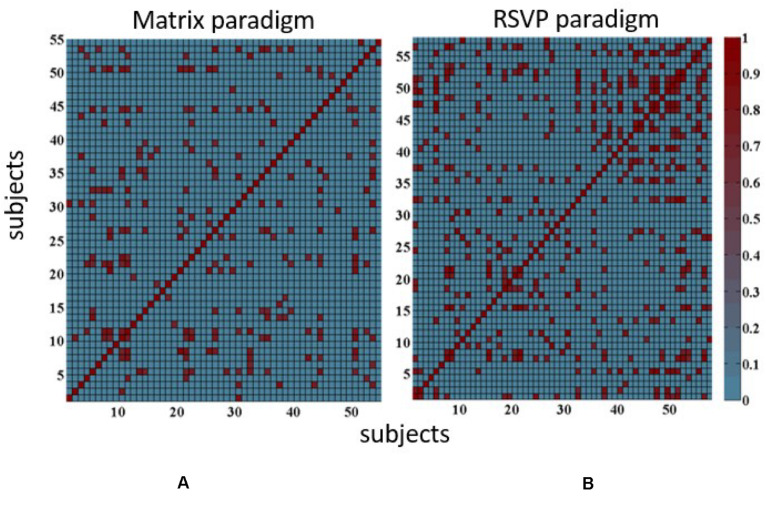
Statistical results of angle cosine values for **(A)** matrix paradigm and **(B)** RSVP paradigm.

To evaluate the similarity of the ERP waveforms from different subjects, the cosine similarity was computed for RSVP and matrix paradigms. According to the calculation principle of cosine similarity, each subject did a correlation analysis with the rest. Figure 6 depicted the individual similarity when the threshold was set to 0.5 for example. That is, when the angle cosine value between two subjects was larger than 0.5, the similarity value was regarded as 1; otherwise, the value was set to 0. As can be seen, the RSVP paradigm had more similarity pair than the matrix paradigm, indicating that ERPs induced by RSVP had less individual difference.

As can be seen in Figure 6, the number of matches for each subject (the number of the included angle cosine values greater than 0.5) was counted; the average matching number of all subjects was 6 for the matrix paradigm, while for the RSVP paradigm, the average matching number range was 20. Especially 12 subjects had more than 20 matched numbers, 3 subjects had more than 30 matched numbers, and the best was 41 for the RSVP paradigm, which meant that the P300 signal waveform induced by the RSVP paradigm was relative to that induced by the matrix paradigm. A smaller inter-individual difference was generated and was more advantageous for cross-subject classification identification.

### ITR Analysis

**FIGURE 7:**
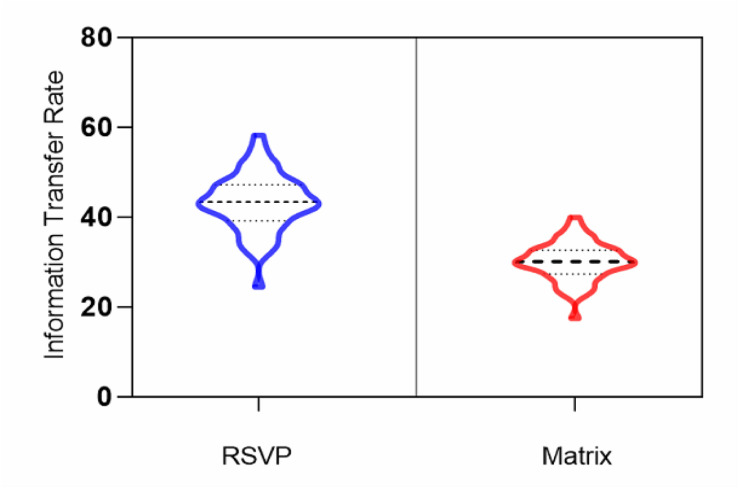
ITR results for the RSVP and matrix paradigms.

Figure 7 displays the ITR differences between the RSVP and matrix paradigms, which were most commonly applied in the BCI community as an important metric to gauge BCI performance. As can be seen, the average information transfer rate of the RSVP paradigm was 43.18 bits/min, while the average value of the matrix paradigm was 30 bits/min. Moreover, the maximum ITR of the RSVP paradigm was 58 bits/min and the minimum was 24 bits/min, and the maximum value of the matrix paradigm was 38 bits/min and the minimum value was 17 bits/min, which proved that the RSVP paradigm was capable of producing a larger information transfer rate, because the RSVP paradigm did not require eye movement, avoiding longer cognitive periods caused by eye movements, which could have a better performance in ERP-based BCI.

### CAR Analyses

The intersubject and cross-subject classification performances were computed, respectively, and the average AUC was about 0.83 for the intersubject condition. When the training and the test samples were extracted from two different subjects, the AUC value drooped to 0.51, indicating that cross-subject classification could significantly reduce the classifier performance *(p* < 0.05).

**FIGURE 8:**
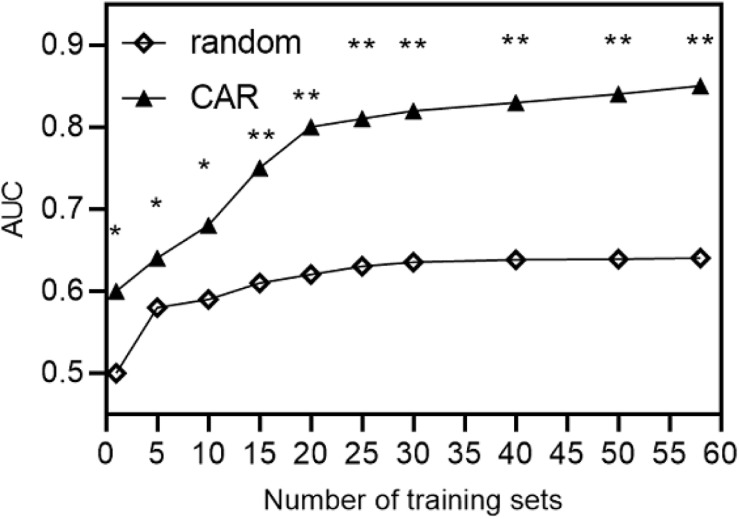
The AUC results of random and order models.

In the cross-subject classification, we tried to increase the number of subjects in the training set to reduce the impact of individual differences on the recognition results. The data from N subjects were randomly selected to form a new training set, and each subject was considered as a test set once termed as the random model. The AUC values in the random model are shown in Figure 8. The transverse coordinates represented the number of subjects in the training set; the longitudinal coordinates represented the AUC values on cross-subject recognition. It can be seen that with the increase in the subjects’ number in the training set, the AUC values increased and the best value was 0.6.

Then a CAR algorithm was proposed to further improve the cross-subject classification. To evaluate the effectiveness of the CAR algorithm, the AUC values were computed accordingly, which are shown in Figure 8. It is obvious that for all N’s in transverse coordinates, the performance of the CAR algorithm was significantly better than that of the order model. Kolmogorov–Smirnov test was used to test the normal distribution of the data. The results showed that the data are in accord with the normal distribution and complied with the *t*-test requirements. So paired *t*-test was used for statistical analysis, and whatever the value of N was, the CAR algorithm could significantly improve the cross-subject performance (*p* < 0.05). With the increase in the subject number with the same sample size in the training set, the AUC value tended to be stable after increasing, and then decreased, and very few test set individuals fluctuated up and down. Moreover, the paired *t*-test was used and showed that whatever the value of N was, there were significant differences between the two models (*p* < 0.05). These encouraging results suggested that with proper evoked paradigm and classification methods, it was feasible to get stable and better performance across subjects for ERP-based BCI.

## Discussion

This study designed an RSVP-based experiment and asked 58 subjects to perform an experiment to investigate cross-subject ERP-based BCI classification methods. The results of the data analysis demonstrated that the RSVP paradigm can evoke smaller individual differences. Moreover, this study proposed a new method to use CAR algorithms to improve the cross-subject ERP-based BCI performance, which has good practical significance, but there are still a lot of problems that need improving.

In the paradigm design, the alphabet order increases the ability of the subjects, but some adjacent letters are more similar (such as E and F), which brings difficulties for the observation and errors in the data classification. Although some effects are eliminated by using the different colors in different letters, still some improvements are needed. In the next attempt, we can mix the uppercase and lowercase letters (such as e and F) to increase the discriminability and visual stimulus. We can also change the presentation times and the size of letters, and add numbers to the stimulus sequence, to analyze the influence of these parameters on cross-subject recognition and explore a more effective general model. Moreover, in this paper, the CAR algorithm is used to improve the performance in cross-subject ERP-based BCI, which has a better result than traditional random selection, but it still needs to establish a large feature database, and this can consume energy and increase the trial time for the subjects. However, the state of the subjects directly affects the quality of signal acquisition. So it is necessary to control the dynamic stop policy experiment by setting the number of attempts. Meanwhile, this study only analyzes and calculates the offline data. It is hoped that the design and experiment in an online environment can be carried out on the basis of this algorithm. At this time, it is necessary to consider the flow design about the online experiment, so that it can take into account the body state and the fatigue degree of the subjects while collecting enough data that can be used for classification.

Moreover, by studying the history of the spellers, we found that most work done in this field was presented in a matrix structure (Lees et al., 2018, 2020; Mijani et al., 2019). In these protocols, the subject should look at different points on the screen during the experiment. Therefore, patients with visual impairment confront the problem using this type of spellers. RSVP systems (Ratcliffe and Puthusserypady, 2020) were proposed to solve this problem, but the defect of these protocols was the long duration of the experiment time that deteriorates the ITR and lowers accuracy. Many scholars have done extensive research on improving the performance of BCI, such as creating a P300 speller performance predictor based on RSVP multifeature (Won et al., 2019), finding the optimal features (Yin et al., 2015, 2016; Won et al., 2018), and using deep neural network algorithm (Zhao et al., 2020), which cannot have a better result in a large subject study. In our study, the average ITR of the RSVP speller is 43.18 bits/min, which is 13% higher than the matrix paradigm, and we achieved the better accuracy results at present in a large subject study.

Last but not least, in this article, we collected 64-electrode EEG signals, but only selected six channel data (FZ, CZ, PZ, O1, O2, OZ) related to visual stimuli for the analysis, which got good offline results. At present, the study is still in the laboratory research exploration stage, and follow-up work will focus on practical development, such as use at home and so on. It is worth mentioning that we used six electrodes’ data and a CAR algorithm in the analysis; it is of great significance to design simple and efficient products in the future that can push forward to exploring the direction of practicality and improving the performance in BCI systems.

In a word, a new generation about BCI technology can be realized by different technical ways in the future, which can maintain high reliability and high stability for different subjects, different times, and different scenarios. This study provides a research idea called the CAR algorithm for the implementation in a cross-subject BCI system and has very important research significance.

## Conclusion

In this paper, the P300 signal was induced by the RSVP paradigm, which does not need eye movement, and we collected the EEG data of 58 subjects. Compared with the matrix paradigm, it is proved that the P300 signal induced by the RSVP paradigm had smaller individual differences, and the AUC value, which represented the performance of the classifier, was used as the evaluation index to analyze the within and cross-subject BCI systems. The results showed that RSVP could evoke more similar ERP patterns among subjects and has a higher ITR compared with the matrix paradigm. Then, the included angle cosine was calculated and counted for the averaged ERP waveform between two subjects, and the threshold value was set to 0.5. The average matching number of all subjects was 6 for the matrix paradigm, while for the RSVP paradigm, it was 20, which was more than three times larger, quantificationally indicating that ERP waveforms evoked by the RSVP paradigm produced smaller individual differences, which is more favorable for cross-subject classification and recognition. ROC values were computed and compared using CAR and traditional random selection. The results showed that the proposed CAR got significantly better performance than traditional random selection, and got the best AUC value of 0.83, while traditional random selection only achieved 0.65. These encouraging results suggest that with a proper evoked paradigm and the CAR algorithm, it is feasible to get stable performance across subjects for ERP-based BCI.

## Data Availability Statement

The raw data supporting the conclusions of this article will be made available by the authors, without undue reservation, to any qualified researcher.

## Ethics Statement

Before the experiment, the experimental procedure was fully explained to each participant, and all participants signed written consent forms. After the experiment, we paid for the participants. The experiment was conducted with approval from the ethics committee of Tianjin University Tianjin hospital.

## Author Contributions

SL, WW, and DM conceptualization. WW: methodology, writing – original draft preparation, and visualization. WW and YS: data collection. YS: software. SL, WW, and YS: validation. LZ: formal analysis, supervision. MX: investigation. DM: resources and funding acquisition. SL: data curation and writing – review and editing. SL and DM: project administration. All authors contributed to the article and approved the submitted version.

## Conflict of Interest

The authors declare that the research was conducted in the absence of any commercial or financial relationships that could be construed as a potential conflict of interest.
